# Evolution of the Cytolytic Pore-Forming Proteins (Actinoporins) in Sea Anemones

**DOI:** 10.3390/toxins8120368

**Published:** 2016-12-08

**Authors:** Jason Macrander, Marymegan Daly

**Affiliations:** 1 Department of Evolution, Ecology, and Organismal Biology, Ohio State University, 1315 Kinnear Rd, Columbus, OH 43212, USA; daly.66@osu.edu; 2 Department of Biological Sciences, University of North Carolina, Charlotte, 9201 University City Blvd., 373 Woodward Hall, Charlotte, NC 282233, USA

**Keywords:** Cnidaria, toxin, target recognition, cytolysins

## Abstract

Sea anemones (Cnidaria, Anthozoa, and Actiniaria) use toxic peptides to incapacitate and immobilize prey and to deter potential predators. Their toxin arsenal is complex, targeting a variety of functionally important protein complexes and macromolecules involved in cellular homeostasis. Among these, actinoporins are one of the better characterized toxins; these venom proteins form a pore in cellular membranes containing sphingomyelin. We used a combined bioinformatic and phylogenetic approach to investigate how actinoporins have evolved across three superfamilies of sea anemones (Actinioidea, Metridioidea, and Actinostoloidea). Our analysis identified 90 candidate actinoporins across 20 species. We also found clusters of six actinoporin-like genes in five species of sea anemone (*Nematostella vectensis*, *Stomphia coccinea*, *Epiactis japonica*, *Heteractis crispa*, and *Diadumene leucolena*); these actinoporin-like sequences resembled actinoporins but have a higher sequence similarity with toxins from fungi, cone snails, and *Hydra*. Comparative analysis of the candidate actinoporins highlighted variable and conserved regions within actinoporins that may pertain to functional variation. Although multiple residues are involved in initiating sphingomyelin recognition and membrane binding, there is a high rate of replacement for a specific tryptophan with leucine (W112L) and other hydrophobic residues. Residues thought to be involved with oligomerization were variable, while those forming the phosphocholine (POC) binding site and the N-terminal region involved with cell membrane penetration were highly conserved.

## 1. Introduction

Like all cnidarians, sea anemones (order Actiniaria) use a complex repertoire of toxic peptides in conjunction with stinging cells called nematocytes to defend against predators and aid in prey capture. Their diverse assemblage of proteinaceous toxins includes phospholipases, neurotoxins, protease inhibitors, and pore-forming toxins [1]. Pore-forming toxins are widespread in Cnidaria and include membrane attack complex component/perforin (MACPF) toxins [2], phospholipase-like cytolysins [3], fire coral cytotoxins [4], hydralysins [5,6], box jellyfish cytotoxins [7], and actinoporins [8]. Among these, actinoporins remain the best studied group and are found exclusively in sea anemones. Actinoporins are highly lethal to small crustaceans, molluscs, and fishes and induce cellular lysis through a multistep process that involves the recognition of sphingomyelin, a sphingolipid in animal cell membranes, prior to pore formation [8,9,10]. 

Actinoporins are comprised of a single domain (~20 kDa), lack cysteine residues, and are equipped with functionally important regions conserved throughout the toxin gene family [11,12,13]. Although actinoporins are presumed to be found only in sea anemones, they resemble peptides from other cnidarians, molluscs, crustaceans, vertebrates, fungi, and plants [8,14,15]. The non-venomous function of actinoporin-like peptides remains unknown in most species; however, in bryophytes they are involved in drought tolerance [16], and in fishes they are presumed to be involved in membrane binding [17]. Among Cnidaria, actinoporin-like toxins have been described in only one non-actiniarian species, *Hydra magnipapillata* [18]. The actinoporin-like toxins in *Hydra* are located within the nematocysts and behave like toxins, but do not target sphingomyelin and exhibit low sequence similarity (~30% identity) to the actinoporins of sea anemones [18,19]. Even when actinoporin-like peptides have low levels of sequence similarity to true actinoporins, they are very similar in structure [20].

Actinoporins have been used to elucidate cell membrane dynamics and to investigate pharmaceutically relevant biomedical applications [21,22,23,24]. Several residues have been manipulated to identify functionally important regions within the protein [25], revealing an aromatic-rich region that forms the phosphocholine (POC) binding site, with a single amino acid residue (W112 in Equinatoxin II (EqII)) taking on a key role in initiating sphingomyelin recognition and pore formation [11,26,27]. Although events leading to oligomerization remain uncertain, both the RGD domain (R144, G145, and D146 in EqII) and a single valine residue (V60 in EqII) are thought to direct protein attachment and play a key role in this process [25,28]. Ultimately, a key hydrophobic arginine (R31 in EqII) and other hydrophobic residues in the α-helix at the N-terminal region are involved in cell membrane penetration and the formation of the ion conductive pathway [29,30,31,32,33,34], forming a selective pore from four monomers [12,35,36], although oligomerizations involving eight or nine peptides have also been proposed [37,38].

Variation in actinoporins has been hypothesized to play a role in prey capture or defense for sea anemones [8]. However, functional variation has been explored in a taxonomically restrictive manner, focusing primarily on EqII from *Actinia equina* (see [27,35,39,40]), and comparative analysis of species-specific isoforms of actinoporins have identified little variation among gene copies [15,41]. We revisit the question of variation in actinoporins by screening genome and transcriptome data of 25 species across four superfamilies. Our combined bioinformatic and phylogenetic methods provide the necessary framework to determine: (1) if functionally important residues are maintained across candidate actinoporins; (2) how actinoporins have evolved across sea anemones; and (3) how actinoporins are related to actinoporin-like proteins from venomous and non-venomous taxa.

## 2. Results

### 2.1. Actinoporin Alignment and Tree Reconstruction

**Figure 1:**
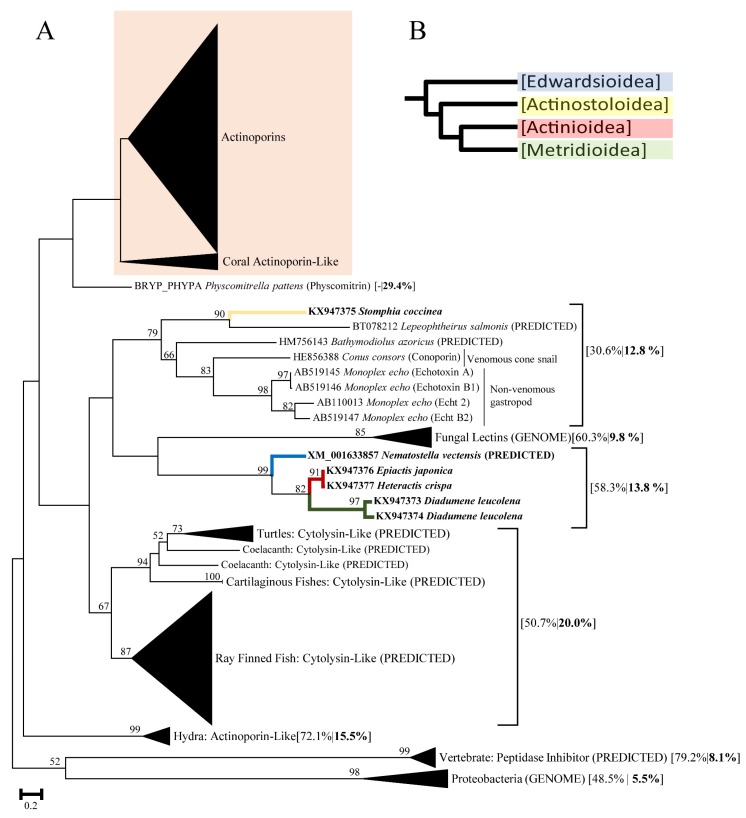
(**A**) Maximum Likelihood tree of actinoporins and actinoporin-like proteins produced in FastTree2. Numbers on branches represent bootstrap values of 1000 replicates. Bootstrapping values greater than 50 are shown at the nodes. Branch labels of clustered gene groups represent lineage common names followed by percent identity (identical amino acid residues) within gene cluster and percent identity when compared to actinoporins in bold. Labeled individual branches show GenBank accession followed by species, and protein name (if applicable). Labels denoted with PREDICTED or GENOME indicate that they were derived bioinformatically in GenBank and are not validated proteins. Branch labels with Genbank accession and species for sea anemones are indicated in bold. The colored box indicates which actinoporin sequences were used in subsequent analyses; (**B**) Phylogenetic tree with branch colors depicting superfamily associations (Blue: Edwardsioidea, Yellow: Actinostoloidea, Red: Actinioidea, Green: Metridioidea) [42].

**Figure 2:**
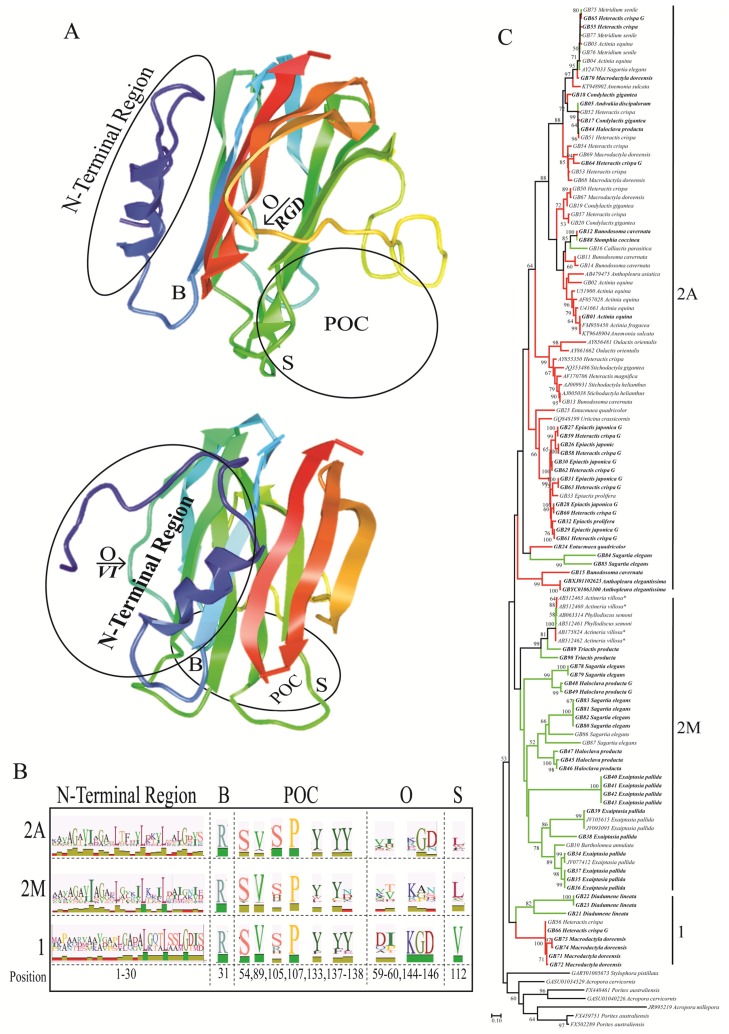
(**A**) Functionally important residues identified on EqII [43]. Functional sites are as follows: (B) site of bend when N-terminus comes into contact with the cell membrane, (POC) residues involved with the POC binding site, (O) residues involved with oligomerization, (S) key sphingomyelin binding site. Colors are used to aid in determining the orientation between the two views shown; (**B**) Characterization of amino acid variation for the different gene clusters (Clade 1, Clade 2M, Clade 2A) identified in our analysis. SeqLogo graphs for residues that have been identified previously as functionally important above the alignment with the size of each amino acid residue representing the frequency in which these residues occurred in the alignment. Numbers along the bottom correspond to positions of specific amino acid residues in EqII; (**C**) Maximum Likelihood actinoporin gene tree produced in FastTree2. Colored branches depict superfamily associations (see Figure 1, Yellow: Actinostoloidea, Red: Actinioidea, Green: Metridioidea). Bootstrapping values greater than 50 are shown at the nodes. Branch labels include GenBank ID (when applicable) and the species from which the toxin gene was derived. Bold labels indicate that the mature protein sequence was recovered. Sequences derived from genomic data are indicated with “G” following species in sequence IDs. The superfamily association for *Actineria villosa* may be incorrect and is noted with an asterisk.

In total we identified genes for 90 actinoporin and six actinoporin-like candidates. Our tBLASTn search against the publicly available data identified a single gene for an actinoporin-like candidate in *Nematostella vectensis* and in several coral species. Additionally, we identified several actinoporin-like sequences from the transcriptomes and genomes of vertebrates, fungi, and bacteria available on GenBank. Several of the actinoporin-like sequences (Figure 1) and actinoporins (Figure 2) identified in our transcriptomes and genomes revealed significant deviations in isoelectric point (pI) and peptide size ranges from what had previously been described in sea anemones [15]. The three taxa for which we surveyed genomic rather than transcriptomic data (*Epiactis japonica*, *Haloclava producta*, and *Heteractis crispa*) exhibited the largest range in pI values and peptide size (Table 1).

The maximum likelihood tree for actinoporin-like sequences (LogLk = −40,408) revealed distinct gene clusters that separated previously characterized actinoporin toxins from actinoporin-like protein sequences (Figure 1), including functionally characterized proteins from *Hydra* [19], mollusks [14], and fungi [20]. In the gene tree of actinoporin-like sequences are several lineage-specific groups; many transcriptomic sequences from vertebrates are classified based on automated designation in GenBank as “peptidase inhibitor and cytolysin-like,” although these sequences have not been functionally characterized (Figure 1). The candidate actinoporins from sea anemones formed a distinct gene cluster that also includes sequences for candidate actinoporins from several scleractinian coral species (Figure 1). Six potential actinoporin-like sequences from five species of sea anemone (*Nematostella vectensis*, *Stomphia coccinea*, *Epiactis japonica*, *Heteractis crispa*, and *Diadumene leucolena*) fall outside of the cluster containing genes from previously characterized actinoporins. Ultimately, a Bryoporin (from *Physcomitrella patens*) [16] had the highest percent identity with candidate actinoporins (29.4%), while for the actinoporin-like sequences derived from sea anemones and other venomous taxa, the percent identity was substantially lower (Figure 1).

The maximum likelihood tree (LogLk = −26,789) for the candidate actinoporins includes several distinct gene clusters with high support values, many of which appear to be taxonomically restricted (Figure 2). The majority of the candidate actinoporins are resolved in ways that accord with superfamily relationships (see [42]), although some of the candidate actinoporins from *Diadumene lineata* (Metridioidea), *Heteractis crispa* (Actinioidea), and *Macrodactyla doreensis* (Actinioidea) formed distinct gene clusters separate from other taxa in their respective superfamilies (Figure 2). Within the actinoporin sequence alignment, these sequences differ considerably from others in their first ~100 amino acids, but are not notably different elsewhere (see Supplemental File 1). Several actinoporin groupings that were previously identified remained intact [1], with the sequences from species in Actiniidae having the largest expansion among those previously characterized. We did not identify any actinoporins from members of Edwardsioidea despite searching expressed sequence tag (EST) libraries and genomes for *Nematostella vectensis* [48] and *Edwardsia elegans* [49].

The actinoporin sequences fall into two major groups: a small gene cluster with relatively few species each from Actinioidea and Metridioidea (Figure 2, Clade 1) and a large gene cluster that contains a primarily metridioidean subclade (Figure 2, Clade 2M) and a primarily actinioidean subclade (Figure 2, Clade 2A). To avoid over extending our analysis, or making assumptions about candidate cytolysins that have not been characterized, we will refer to these three actinoporin clades, but recognize the low bootstrap values for some nodes and understand that this tree may change with the addition of more taxa. The clade that contains the majority of sequences from members of the superfamily Metridioidea (Figure 2, Clade 2M) also includes two sequences from the actinioidean *Actineria villosa*, which was probably misidentified, resulting in the observed placement within the tree [50]. A few sequences from metridioideans lie outside of this large clade, as the sister to a larger clade comprised of sequences from actinioideans (Figure 2, Clade 1) or nested within the large clade of sequences from Actinioidea (Figure 2, *Andvakia discipulorum*, *Calliactis parasitica*, and *Sagartia elegans* within Clade 2A). The actinoporins that have been functionally characterized are all found in clade 2A, with the exception of those from *Actineria villosa* and *Phyllodicus semoni* [15,34,51].

The clusters within the actinoporin gene tree also bear some signature of the methods used to collect the sequences. In clade 2A, there was a gene cluster comprised primarily of sequences from *Heteractis crispa* and *Epiactis japonica* that were generated through genomic (rather than transcriptomic) methods (Figure 2). The genomic data sets contained more isoforms than the transcriptomic data sets in general, but isoform diversity also seems to have a taxonomic component (Table 1). The actinioidean *Heteractis crispa* has the most isoforms among the species in our analyses (Table 1).

### 2.2. Functionally Important Residues

Several amino acid residues were highly conserved across all candidate actinoporin sequences (see Supplemental File 1). Of the seven amino acid residues forming the POC binding site, four were highly conserved (S54, V87, S105, and P107), with the remaining three (Y133, Y137, and Y138) exhibiting some variation (Figure 2). Although several residues are involved with sphingomyelin recognition [27,52], a single tryptophan (W112 in EqII) has been hypothesized to be an essential residue in pore formation [53]. Three of the previously identified amino acids contributed the majority of the diversity (leucine, tryptophan, and phenylalanine) at this residue, although several amino acid residues not identified at this site previously (lysine, methionine, glycine, valine, serine, and tyrosine) also occur. For sequences in clade 2A, the clade in which function has been characterized most broadly, the majority of sequences had a leucine at this site (27/50), followed by tryptophan (12/50), phenylalanine (9/50), and methionine (2/50) (see Supplemental File 1).

Residues hypothesized to be involved in oligomerization were much more variable than those inferred to act in binding (Figure 2). The RGD domain (R144, G145, and D146) forms a bend in the protein between two β–strands and was hypothesized to maintain correct oligomerization in order to form a functional pore, but may also play an important role in directing protein attachment to some integrin-like receptors [28]. Many of the actinoporin candidates in clade 2A are instead equipped with KGD (R144K) residues, and the actinoporin candidates in clade 2M are equipped with EGD (R144E) residues or have a deletion in this region (Figure 2). Additionally, other residues thought to play a key role in oligomerization (I59 and V60) were highly variable [25] and did not appear to follow any phylogenetic pattern (Figure 2).

**Figure 3:**
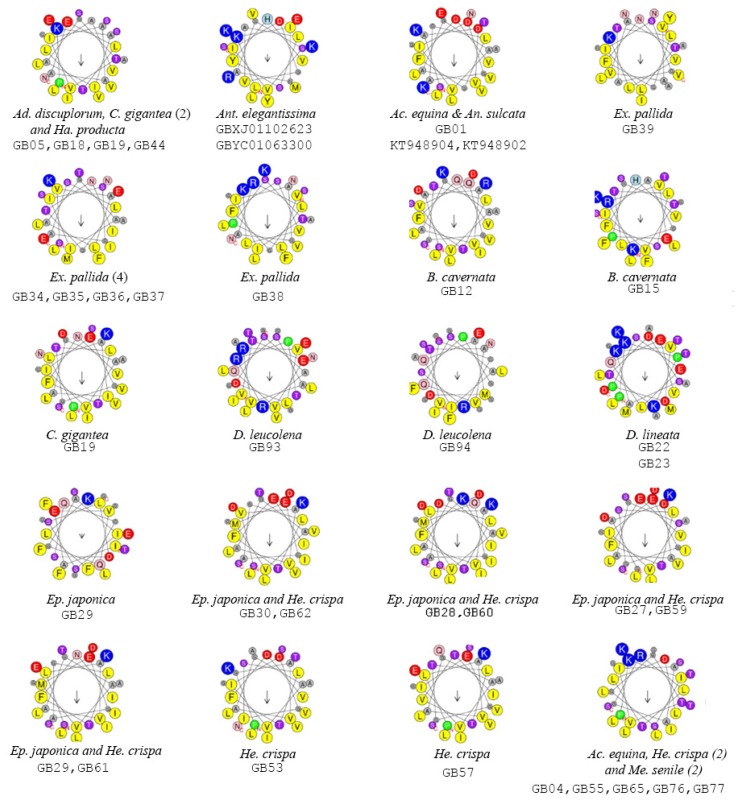

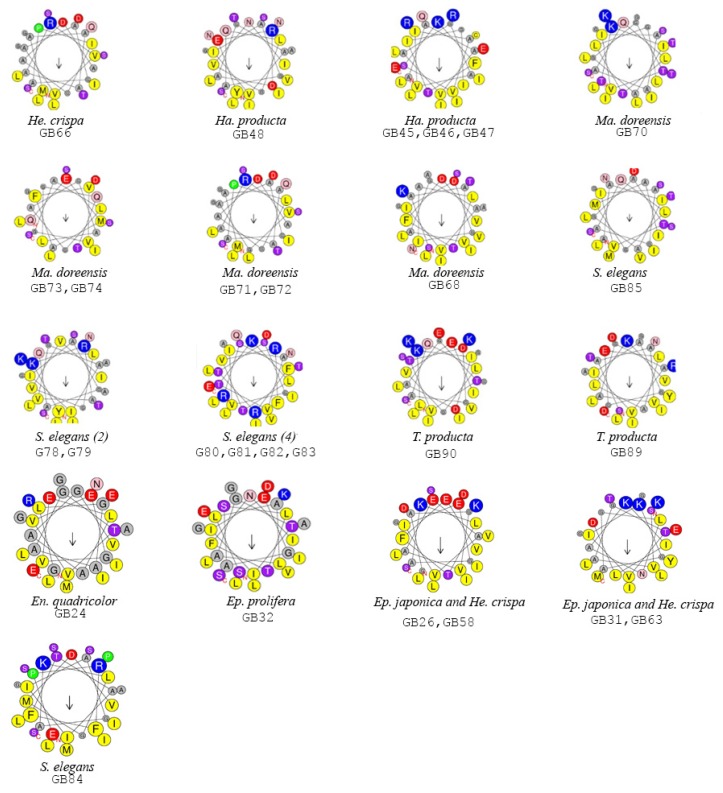
Edmundson wheel projections were determined in HeliQuest. Associated Sequence IDs are shown below the species name.

By combining sequence alignments of previously characterized proteins and predicted protein structure, we were able to identify 64 unique N-terminal regions from candidate actinoporins (Figure 3). The N-terminal region contains approximately 18 amphipathic amino acids which are highly conserved and necessary for α-helix formation and for penetration of the lipid membrane [30,32]. Representative N-terminal regions were screened (26–31 amino acids) in the full window size as the start/stop of the α-helix in the candidate peptides was sometimes unclear (Table 1). Edmundson wheels of potentially unique N-terminal regions within the gene tree are reported (Figure 3). For the majority of these, the net charge is negative (Table 1), likely due to the hydrophobic residues in the α-helix. We found several highly conserved hydrophobic residues (G6, V8, I9, G11, L14, 19L, L23, L26, G27), common to all candidate actinoporins except for some actinoporin candidates from *Exaiptasia pallida* and *Haloclava producta* (see Supplemental File 1). A single arginine residue (R31), responsible for movement of the α-helix into the cellular membrane [33,34,54], was conserved across all actinoporin sequences (Figure 2).

### 2.3. Selection Analyses

Our investigation into rates of selection across the actinoporin tree indicates that these toxin genes have evolved under high rates of negative selection. This inference holds when examining the actinoporins as a whole (ω < 1) and when we focus on specific gene clusters (Table S1). With respect to site-specific rates of evolution across all candidate actinoporins, the Bayes empirical Bayes (BEB) approach failed to identify any positively selected sites. However, when focusing instead on specific gene clusters at lower hierarchical levels, the BEB analysis identified three sites that were significantly under the influence of positive selection, corresponding to the candidate actinoporin gene cluster containing sequences from predominantly actinioideans (Table S1). None of the positively selected sites, however, corresponded with residues of a known functional role. 

## 3. Discussion

Analysis of our transcriptomic and genomic datasets substantially increased the number of candidate actinoporins and the phylogenetic diversity of sea anemones from which they have been reported. Although multiple residues are involved in sphingomyelin recognition [27,52,53], tryptophan (W112 in EqII) has been regarded as a functionally important residue involved in this process [11,26,27,55]. Our results indicate that variation at this site exceeds what was previously proposed, with L112 as the most common residue (Figure 2). Many variants at this location (L, F, Y, M, V, S) are considered the next-best candidates for replacement according to the Wimley and White hydrophobicity scale [56]. Sphingomyelin specificity occurs via hydrogen bonding to the 3-OH and 2-NH groups at the side chains of D109 and Y113 and main chains of P81 and W112 (in EqII); we find that almost every candidate actinoporin sequence was equipped with a P81, D109, Y113 and some hydrophobic variant at position 112; this supports previous hypotheses that although multiple residues are involved with sphingomyelin recognition, the hydrophobic residue at this position can vary and likely remain functional [27,52,53].

Although sequence alignments for candidate actinoporins were relatively conserved (Supplemental File 1), we find significant variation at several residues that are presumed to be functionally important. The aromatic rich region and adjacent POC binding site was variable at three sites (Y133, Y137, and Y138) in several metridioidean candidate actinoporins (Figure 2). Sites involved with oligomerization (the RGD domain and V60) were variable, but this variation was not lineage specific (Figure 2). For most candidate actinoporins, the N-terminal region had many of the essential hydrophobic residues in addition to R31 (conserved across all actinoporins), suggesting that these residues are necessary for pore formation. Collectively, subtle differences across actinoporins are only now being explored in a comparative context [53] and evolutionary history among actinoporins may provide insight into how these proteins function.

We identified six actinoporin-like sequences in sea anemones (Figure 1), which could be ancestral paralogs or proteins under the influence of strong selection pressures, resulting in convergent evolution among structural components shared between these proteins. Within this same portion of the actinoporin-like gene tree there were several actinoporin-like toxins (Figure 1), including *Hydra* actinoporin-like toxins [19], molluscan echotoxins [14], and fungal lectins [20]. Although the edwardsioideans do not appear to have any candidate actinoporins (Figure 2), we identified actinoporin-like sequences in *Nematostella vectensis* and other sea anemones (*Heteractis crispa*, *Epiactis japonica*, and *Diadumene leucolena*); the molecules these encode may still be involved in envenomation and function like actinoporins (Figure 1). Our broad taxonomic approach resulted in lower bootstrap support for actinoporin and actinoporin-like clades, which was less apparent in other actinoporin gene tree reconstructions that were taxonomically limited in comparison [1,19]. Localized expression in actinoporins and actinoporin-like proteins remains unknown, as previous investigations into nematocyst contents failed to recover actinoporins in sea anemone nematocysts [56], although they may simply be expressed in ectodermal glands [57]. Structural similarities between actinoporin-like proteins from sea anemones and other venomous taxa could provide insight into how these proteins behave [19]. Further investigation is needed to properly characterize the actinoporin and actinoporin-like candidates as there are limitations in what we can derive regarding toxin function from transcriptome sequencing alone [58,59].

Our broad taxonomic sampling revealed that actinoporins may also be present in the venom of scleractinian corals and could indicate that this gene family of toxins was recruited in an anthozoan ancestor. The venom components in scleractinian corals are poorly understood [60,61], with no known functional information about these actinoporins. Evolutionary processes shaping the actinoporin gene family is further complicated with no candidate actinoporins being identified in two edwardsioideans included in our study (*Nematostella vectensis* and *Edwardsia elegans*), however there was a single actinoporin-like sequence for *Nematostella vectensis* (Figure 2). The lack of candidate actinoporins, but presence of actinoporin-like sequences within Edwardsioidea could be the result of an ancestral loss of functional venom components, while retaining the ancestral actinoporin-like proteins. The functional role of actinoporin-like proteins in sea anemones remains unclear; however, the presence of actinoporin like-sequences from *Nematostella vectensis* within this paralogous group and not within the larger clade of actinoporins (Figure 1) indicates that the ancestral toxic actinoporin may have been lost in Edwardsioidea.

The candidate toxins from species in Actinioidea and Metridioidea provide significant insight into how the actinoporin genes have evolved in sea anemones. Previous actinoporin gene tree reconstructions formed taxonomically distinct groupings, all of which were recovered to some degree in our analysis. However, a large, strongly supported gene cluster containing sequences from actinioideans (see Figure 4, [1]), now includes several non-actinioidean taxa: *Sagartia elegans* (see [62]), *Metridium senile*, *Andvakia discipulorum*, *Calliactis parasitica*, and *Stomphia coccinea* (Figure 2). Sister to this gene cluster is a strongly supported gene cluster of newly identified candidate actinoporins, determined from primarily genomic data from *Heteractis crispa* and *Epiactis japonica* (Figure 2). Several other candidate actinoporins (from the metridioideans *Exaiptasia pallida*, *Haloclava producta*, and *Sagartia elegans*) formed species-specific gene clusters, with each cluster appearing to follow a pattern of concerted evolution (Figure 2).

Although there have been significant advances in characterizing the functionally important residues across actinoporins, the role of actinoporins in sea anemone venom is perplexing. Actinoporins have not been identified in nematocysts [56,63], although genetic components (signal peptide and propart motif) indicate that they are likely synthesized in the Golgi apparatus during nematocyst development [15,64]. Additionally, tissue-specific studies have shown that actinoporins are expressed in mesenterial filaments, suggesting that they play a role in digestion [65]. Despite most prey or predator species having a relatively conserved and ubiquitous actinoporin target site (sphingomyelin), many highly conserved actinoporins exhibit variable rates of cytolytic activities [40]. This suggests that minor changes in amino acids across actinoporins may have co-diversified to target cell membranes in specific lineages [8,15,41,65,66]. There is still much to be done to understand how these venoms function in an ecological and evolutionary context.

## 4. Materials and Methods

### 4.1. Partial Transcriptome and Genome Sequencing and Assembly

Transcriptomes and genomes from 25 species of sea anemones (*Nematostella vectensis* [48], *Edwardsiella lineata* [49], *Stomphia coccinea*, *Actinia equina*, *Anemonia sulcata* [44], *Anthopleura elegantissima* [46], *Bunodosoma cavernata*, *Condylactis gigantea*, *Entacmaea quadricolor*, *Epiactis japonica*, *Epiactis prolifera*, *Macrodactyla doreensis*, *Heteractis crispa* [44], *Bartholomea annulata*, *Exaiptasia pallida* [47], *Andvakia discipulorum*, *Diadumene leucolena*, *Diadumene lineata*, *Calliactis polypus*, *Haloclava producta*, *Metridium senile*, *Sagartia elegans*, and *Triactis producta*) were used to identify candidate cytolysins and cytolysin-like sequences. The unpublished transcriptomes’ RNA isolation and sequencing were carried out similar to what was published previously [44,46]. In brief, specimens were frozen in liquid nitrogen or placed in RNALater (QIAGEN, Hilden, Germany) immediately after being collected. For a comprehensive list of sequencing methods and tissues used for focal taxa, see Table S2. Tissues or whole specimens were placed in a 2 mL screwcap vial (BioExpress, Kaysville, UT, USA) with 600 µL of Buffer RLT from the RNeasy isolation kit (QIAGEN, Hilden, Germany). Several (4–8) small (1.5–2 mm) ceramic beads (BioExpress, Kaysville, UT, USA) were added to each vial and the specimen or tissue was macerated using a Mini-Beadbeater-8 (BioSpec Products, Bartlesville, OK, USA) for 30 s at the “Homogenize” setting. After 30 s, the vial was visually inspected to determine if additional homogenization was necessary. Total RNA was extracted using the RNeasy Kit (QIAGEN, Hilden, Germany). The first strand synthesis was done using RNA using the TruSeq Stranded mRNA LT Sample Prep Kit (Illumina, San Diego, CA, USA) and sequencing completed at the Nucleic Acid Shared Resource—Illumina Core (The Ohio State University, Columbus, OH, USA) on an Illumina Hiseq 2500. The genomic DNA was extracted using the DNeasy Kit (QIAGEN, Hilden, Germany) with the library construction (2 × 300 bp) and sequencing carried out at the Ohio Agricultural Research and Development Center (The Ohio State University, Wooster, OH, USA) on the Illumina Mi-Seq. The raw reads from both transcriptome and genome sequencing runs were subjected to an error correction step in the program Trimmomatic, vers. 0.36 [67]. Transcriptomes were assembled de novo in the assembly program Trinity, vers. 2.1.1 [68]; genomes were assembled using DISCOVAR de novo vers. r52325 [69], followed by a second de novo assembly in Geneious, vers. R7, Auckland, New Zealand [70]. 

### 4.2. Identification of Actinoporin and Actinoporin-Like Sequences

Protein sequences from previously characterized actinoporins were downloaded from UNIPROT (Table S3) and used to identify candidate actinoporin sequences in our transcriptome and genome data sets using tBLASTn [71]. Transcripts identified in the BLAST search with an *E*-value greater than 0.001 were further evaluated on overall sequence alignment. Additional genes were identified in subsequent tBLASTn searches against the nucleotide database in GenBank, using the newly identified sea anemone candidate actinoporins as query sequences. Each of the identified actinoporin and actinoporin-like candidate transcripts were trimmed to include only the open reading frame and to exclude the stop codon. Complete and partial sequences were included in our analyses (Supplemental File 2). Candidate actinoporin and actinoporin like sequences are available on Genbank (KX947295-KX947298, KX947301-KX947377).

Two gene trees were constructed to distinguish candidate actinoporins from actinoporin-like sequences. The first gene tree included the 248 protein sequences that were translated from their nucleotide sequence (Table S4). Protein sequences were aligned in MAFFT, vers. 7.017 [72] in Geneious, vers. R7, Auckland, New Zealand with the G-INS-i algorithm, BLOSUM 62 scoring matrix, and a gap open penalty of 1.53 and offset value of 0.123 (Supplemental File 1). A maximum likelihood tree was reconstructed using FastTree, vers. 2.1 [73] under the WAG model with 1000 bootstrapping replicates resampled in PHYLIP’s SEQBOOT program, vers. 3.695 [74]. Isoelectric point (pI) and molecular weight were calculated for candidate actinoporins using ExPASy [75].

The combined tree of actinoporin and actinoporin-like protein sequences was used to determine which sequences could be classified as candidate actinoporins (Figure 1). The large gene cluster (sea anemones and corals) exclusively containing sequences resembling actinoporins that have been functionally characterized were considered candidate actinoporins. The sequences from sea anemones that were found outside this cluster were considered actinoporin-like. 

A second tree was reconstructed using only nucleotide sequences from the candidate actinoporin gene cluster. Nucleotide sequences from candidate actinoporins were aligned in MAFFT , vers. 7.017 [72] using the translation alignment in Geneious, vers. R7, Auckland, New Zealand [70] with the G-INS-i algorithm, BLOSUM 80 scoring matrix, and a gap open penalty of 1.53 and offset value of 0.123 (Supplemental File 2). Maximum likelihood trees were reconstructed in FastTree, vers. 2.1 [73], using the GTR model with 1000 bootstrapping replicates being resampled in PHYLIP’s SEQBOOT program [74].

### 4.3. Evaluation of Functionally Important Residues

Sequence alignments that included previously characterized actinoporins were used in combination with predicted protein structures to identify residues that might have functional importance in sphingomyelin recognition, oligomerization, or pore formation. The alignments were also used to visualize the proportion of each amino acid residue using SeqLogo in Geneious, vers. R7, Auckland, New Zealand [70]. Functionally important amino acid residues were inspected in the multiple sequence alignments to characterize variation in candidate actinoporins and to determine whether the variants were functionally equivalent replacements or whether they corresponded to phylogeny. Protein structure for candidate actinoporin sequences were predicted in the program Geneious, vers. R7, Auckland, New Zealand [70] using the EMBOSS, vers. 6.5.7 Garnier tool [76].

The N-terminal region of candidate actinoporins was screened for the presence of an α-helix, which initiates the membrane integration and pore formation. As the size of the mature peptide α-helix region varies considerably, we used both conserved residues and predicted tertiary structure to identify potential functionally important residues within the sequence alignments [15,77]. Sequences were trimmed to include approximately the first 30 residues after the potential cleavage site (LL or LR residues) as estimated in SignalP, vers 4.1 [78]. Mean hydrophobicity (<H>), mean hydrophobic moment (<μH>), and Edmundson wheel projections were determined in HeliQuest, vers. 2.0 [79].

### 4.4. Selection Analyses

Rates of selection were determined for the actinoporin gene tree via maximum likelihood models in the program CODEML in PAML, vers. 1.3.1 [80]. A Likelihood-Ratio Test (LTR) was used to perform a series of pairwise comparisons for alternative models of selection. The comparisons performed include: (1) rates across all sites to determine if ω was constant (null model, M0) versus varied (sites grouped into discrete categories based on ω rates, M3); (2) rate of selection across sites indicating nearly neutral evolution (negative ω < 1 or neutral ω = 1, M1) versus positive selection (positive ω > 1, M2); and (3) absence of positively selected sites (positive selection and ω > 1, M7) versus presence of positively selected sites (positive selection and ω < 1, M8). Amino acids under positive selection were identified using the Bayes empirical Bayes (BEB) approach by calculating the posterior probabilities that a particular amino acid belongs to a given selection class (neutral, conserved, or highly variable).

## Figures and Tables

**Table 1:** Candidate actinoporin information and N-terminal helix properties.

Superfamily/*Species*	N	pl	MW (kDa)	(<H>) *	(<μH>) *	Net Charge
**Actinioidea**	**52**					
*Actinia equina*	4	9.47	21.6	0.424	0.337	−2
*Anemonia sulcata* [44,45]	2	9.50	21.7	0.424	0.337	−2
*Anthopleura elegantissima* [46]	2	8.63–8.64	21.2–21.3	0.443	0.207	2
*Bunodosoma cavernata*	5	9.23–10.65	20.4–21.0	0.482	0.356	0
				0.586	0.21	2
*Condylactis gigantea*	4	4.56–6.71	21.57–21.58	0.589	0.35	−1
*Entacmaea quadricolor*	2	5.87	21.8	0.472	0.309	−3
*Epiactis japonica*	6	4.86–9.38	21.4–25.2	0.494	0.122	−2
				0.58	0.336	−3
				0.592	0.363	−1
				0.42	0.361	−4
				0.59	0.342	−2
				0.474	0.396	1
*Epiactis prolifera*	2	8.00	21.5	0.614	0.366	−2
*Heteractis crispa* [44]	17	4.71–9.38	19.5–21.2	0.599	0.351	−1
				0.616	0.288	−1
				0.567	0.327	2
				0.442	0.288	−1
*Macrodactyla doreensis*	8	5.72–7.75	19.1–21.6	0.629	0.287	2
				0.523	0.202	−2
				0.398	0.249	−1
				0.615	0.328	−1
**Metridioidea**	**37**					
*Andvakia discipulorum*	1	4.63	21.6	0.583	0.322	−1
*Bartholomea annulata*	1	-	-	-	-	-
*Calliactis parasitica*	1	-	-	-	-	-
*Diadumene lineata*	3	8.72	23.4	0.211	0.245	−1
						
*Exaiptasia pallida* [47]	10	9.45–10.26	21.7–23.0	0.64	0.268	1
				0.605	0.383	3
				0.556	0.319	−1
*Haloclava producta*	6	4.51–9.91	21.6–27.2	0.474	0.317	−1
				0.682	0.37	1
*Metridium senile*	3	-	-	-	-	-
*Sagartia elegans*	10	8.33–9.66	21.6–25.3	0.606	0.2	−1
				0.514	0.279	3
				0.533	0.28	2
				0.631	0.325	0
*Triactis producta*	2	8.56–9.7	21.1	0.424	0.386	−1
**Actinostoloidea**	**1**					
*Stomphia coccinea*	1	9.12	18.4	0.481	0.312	−1

N: Number of Actinoporin candidates; pI: Isoelectric point; MW1: Molecular weight; (<H>): Hydrophobicity; (<μH>): Hydrophobic moment; - indicates no data. Actinoporin-like sequences are not shown. Previously published datasets are noted. All other sequences are new to this study. * values only shown for full (complete) candidate actionoporin sequence, no partial sequences.

## Supplementary Materials

The following are available online at www.mdpi.com/2072-6651/8/12/368/s1, Table S1: CodeML outputs for different specific regions of the cytolysin tree. Table S2: Transcriptome and genome information for focal taxa. Table S3: UNIPROT accessions for actinoporin proteins used in the initial BLAST searches. Table S4: Genbank accessions for actinoporin-like sequences used in Figure 1. Supplemental File 1: Protein sequence alignment for actinoporin and actinoporin-like sequences. Supplemental File 2: Nucleotide sequences for candidate actinoporins.
