# Supplementary material for: Evolution of the Cytolytic Pore-Forming Proteins (Actinoporins) in Sea Anemones
Source: Toxins (Basel). 2016 Dec 8;8(12):368. doi: 10.3390/toxins8120368 (PMC5198562; doi:10.3390/toxins8120368)
Supplement: Supplementary file 1 [file toxins-08-00368-s001.zip › toxins-153104-Supplemental for publish.docx]

**Supplementary Materials: Evolution of the Cytolytic Pore-Forming Proteins (Actinoporins) in Sea Anemones**

**Jason Macrander and Marymegan Daly**

**Table S1.** CodeML outputs for different specific regions of the cytolysin tree.

| **All** |  |  |  |  | |  | |
| --- | --- | --- | --- | --- | --- | --- | --- |
| **Model** | **Parameters** | **Likelihood (l)** | **w** | **Parameters** | | **No. of Sites with w > 1** | |
| M0 (One Ratio) | 1 | −11,692.32462 | 0.34 |  | | - | |
| M1 (Neutral) | 2 | −11,498.42095 | 0.43 | p: 0.75727 0.24273 w: 0.25035 1.00000 | | - | |
| M2 (Selection) | 4 | −11,496.458 | 0.47 | p: 0.73924 0.22991 0.03085 w: 0.25697 1.00000 1.62620 | | 2 (PP ≥ 0.99)  0 (PP ≥ 0.95) | |
| M3 (Discrete) | 5 | −11,416.67755 | 0.38 | p: 0.30481 0.51727 0.17792 w: 0.10532 0.34244 0.94209 | | - | |
| M7 (beta) | 2 | −11,404.73306 | 0.37 | p = 0.88485 q = 1.47248 | | - | |
| M8 (beta & w) | 4 | −11,396.34995 | 0.38 | p0 = 0.92361 p = 1.14972  q = 2.54768 (p1 = 0.07639) w = 1.18605 | | 0 (PP ≥ 0.99)  1 (PP ≥ 0.95) | |
|  |  |  |  |  | |  | |
| LRT | M0 vs M3 | 551.294136 |  | <<0.001 | |  | |
|  | M1 vs. M2 | 3.925896 |  | >0.05 | |  | |
|  | M7 vs M8 | 16.766224 |  | >0.05 | |  | |
| **Top Cluster (Mostly Actinioidea)** | | | | | | | |
| **Model** | **Parameters** | **Likelihood (l)** | **w** | | **Parameters** | | **No. of Sites with w > 1** |
| M0 (One Ratio) | 1 | −3827.848439 | 0.4415 | |  | | - |
| M1 (Neutral) | 2 | −3681.783062 | 0.486 | | p: 0.58837 0.41163 w: 0.12690 1.00000 | | - |
| M2 (Selection) | 4 | −3671.268264 | 0.578 | | p: 0.57664 0.38118 0.04218 w: 0.13072 1.00000 2.88270 | | 2 (PP ≥ 0.99)  0 (PP ≥ 0.95) |
| M3 (Discrete) | 5 | −3670.187972 | 0.515 | | p: 0.52624 0.27335 0.20041 w: 0.10374 0.61105 1.46155 | | - |
| M7 (beta) | 2 | −3679.062086 | 0.427 | | p = 0.40388 q = 0.54212 | | - |
| M8 (beta & w) | 4 | −3668.873722 | 0.513 | | p0 = 0.81659 p = 0.59590  q = 1.41599 (p1 = 0.18341) w = 1.48698 | | 2 (PP ≥ 0.99)  1 (PP ≥ 0.95) |
| LRT | M0 vs M3 | 315.320934 | 4 | | <<0.001 | |  |
|  | M1 vs. M2 | 21.029596 | 2 | | >0.05 | |  |
|  | M7 vs M8 | 20.376728 | 2 | | >0.05 | |  |

**Table S1.** *Cont.*

| **Bottom Cluster (Mostly Metridioidea)** | | | | | |
| --- | --- | --- | --- | --- | --- |
| **Model** | **Parameters** | **Likelihood (l)** | **w** | **Parameters** | **No. of Sites with w > 1** |
| M0 (One Ratio) | 1 | −5072.816761 | 0.4592 |  | - |
| M1 (Neutral) | 2 | −4954.893361 | 0.611 | p: 0.43880 0.56120 w: 0.11424 1.00000 | - |
| M2 (Selection) | 4 | −4951.627788 | 0.696 | p: 0.42188 0.53432 0.04381 w: 0.11035 1.00000 2.63678 | 0 (PP ≥ 0.99)  0 (PP ≥ 0.95) |
| M3 (Discrete) | 5 | −4942.819067 | 0.588 | p: 0.34921 0.51119 0.13960 w: 0.05939 0.62129 1.57532 | - |
| M7 (beta) | 2 | −4947.400943 | 0.5 | p = 0.42554 q = 0.42725 | - |
| M8 (beta & w) | 4 | −4943.756405 | 0.565 | p0 = 0.93289 p = 0.46616  q = 0.52980 (p1 = 0.06711) w = 1.91486 | 0 (PP ≥ 0.99)  0 (PP ≥ 0.95) |
| LRT | M0 vs M3 | 259.995388 | 4 | <<0.001 |  |
|  | M1 vs. M2 | 6.531146 | 2 | >0.05 |  |

Table S2. Focal taxa (published and unpublished) with transcriptome information used in this study. * Superfamily designation may be incorrect [1]. A dash indicates missing data.

| **Superfamily [2]** | **Family** | **Species** | **Tissues** | **Sequencing** | **CEGMA** |
| --- | --- | --- | --- | --- | --- |
| Edwardsioidea | Edwardsiidae | *Nematostella vectensis* | *NA* | [3] | NA |
| Edwardsioidea | Edwardsiidae | *Edwardsiella lineata* | *NA* | [4] | NA |
| Actinostoloidea | Actinostolidae | *Stomphia coccinea* | T, F | RNA: PE-100 | 99.19% |
| Actinioidea | Actiniidae | *Actinia equina* | T | RNA: PE-100 | 96.77% |
| Actinioidea | Actiniidae | *Anemonia sulcata* | T, F, C | RNA: PE-100 [5] | 94.76% |
| Actinioidea | Actiniidae | *Anthopleura elegantissima* | Acrorhagi | RNA: PE-100 [6] | 98.79% |
| Actinioidea | Actiniidae | *Bunodosoma cavernata* | T | RNA: PE-100 | 84.27% |
| Actinioidea | Actiniidae | *Condylactis gigantea* | T | RNA: PE-100 | 97.98% |
| Actinioidea | Actiniidae | *Entacmaea quadricolor* | T | RNA: SE-50 | 61.29% |
| Actinioidea | Actiniidae | *Epiactis japonica* | NA | DNA: PE-300 | 51.61% |
| Actinioidea | Actiniidae | *Epiactis prolifera* | Whole | RNA: SE-50 | 49.60% |
| Actinioidea | Actiniidae | *Macrodactyla doreensis* | T | RNA: PE-100 | 82.26% |
| Actinioidea * | Haloclavidae | *Haloclava producta* | Whole | RNA: PE-100  DNA:PE-300 | 91.53%  - |
| Actinioidea | Stichodactylidae | *Heteractis crispa* | T, F, C | RNA: PE-100 [5]  DNA:PE-300 | 94.35%  - |
| Metridioidea | Aiptasiidae | *Bartholomea annulata* | T | RNA: SE-50 | 75.00% |
| Metridioidea | Aiptasiidae | *Exaiptasia pallida* | *NA* | SRR696721 [7] | 96.37% |
| Metridioidea | Aliciidae | *Triactis producta* | Whole | RNA: PE-100 | 94.35% |
| Metridioidea | Andvakiidae | *Andvakia discipulorum* | Whole | RNA: PE-100 | 78.63% |
| Metridioidea | Boloceroididae | *Bunodeopsis globulifera* | Whole | RNA: PE-100 | 98.79% |
| Metridioidea | Diadumenidae | *Diadumene leucolena* | Whole | RNA: PE-100 | 91.94% |
| Metridioidea | Diadumenidae | *Diadumene lineata* | Whole | RNA: PE-100 | 93.16% |
| Metridioidea | Hormathiidae | *Calliactis polypus* | T, C, | RNA: PE-100 | 89.11% |
| Metridioidea | Metridiidae | *Metridium senile* | T, C, Ac, Ct | RNA: PE-100 | 97.18% |
| Metridioidea | Sagartiidae | *Sagartia elegans* | Whole | RNA: PE-100 | 95.97% |

Table S3. UNIPROT accessions for actinoporin proteins used in the initial BLAST searches.

| **Entry** | **Taxon** |
| --- | --- |
| Q5R231 | *Actineria villosa* |
| D2YZQ3 | *Actineria villosa* |
| P0C1H2 | *Actinia equina* |
| P0C1H1 | *Actinia equina* |
| P0C1H0 | *Actinia equina* |
| P61914 | *Actinia equina* |
| Q9Y1U9 | *Actinia equina* |
| Q93109 | *Actinia equina* |
| B9W5G6 | *Actinia fragacea* |
| P30833 | *Actinia tenebrosa* |
| P30834 | *Actinia tenebrosa* |
| P61915 | *Actinia tenebrosa* |
| A0A0S1M151 | *Anemonia sulcata* |
| A0A0S1M152 | *Anemonia sulcata* |
| A0A0S1M135 | *Anemonia sulcata* |
| C5NSL2 | *Anthopleura asiatica* |
| P0DMX3 | *Entacmaea quadricolor* |
| P58691 | *Heteractis crispa* |
| P0C1F8 | *Heteractis crispa* |
| P58689 | *Heteractis magnifica* |
| P58690 | *Heteractis magnifica* |
| P39088 | *Heteractis magnifica* |
| P0DMX2 | *Heteractis magnifica* |
| Q9U6X1 | *Heteractis magnifica* |
| Q5I4B8 | *Oulactis orientalis* |
| Q5I2B1 | *Oulactis orientalis* |
| P0DL55 | *Phyllodiscus semoni* |
| P0DL56 | *Phyllodiscus semoni* |
| Q86FQ0 | *Sagartia rosea* |
| H9CNF5 | *Stichodactyla gigantea* |
| P07845 | *Stichodactyla helianthus* |
| P81662 | *Stichodactyla helianthus* |
| P0DMX4 | *Stichodactyla mertensii* |
| C9EIC7 | *Urticina crassicornis* |

**Table S4.** Genbank accessions for actinoporin-like proteins used in Figure 1.

| AB063314.1 | GARY01005673.1 | XM_006003820.2 | XM_011321400.1 |
| --- | --- | --- | --- |
| AB110013.2 | GASU01034529.1 | XM_006120076.2 | XM_011327718.1 |
| AB175824.1 | GASU01040226.1 | XM_006134180.1 | XM_011478377.1 |
| AB479475.1 | GBFD01000386.1 | XM_006793231.1 | XM_011604155.1 |
| AB512460.1 | GBFD01006301.1 | XM_006860003.1 | XM_011608329.1 |
| AB512461.1 | GBFD01006728.1 | XM_007052718.1 | XM_011608385.1 |
| AB512462.1 | GBXJ01102623 | XM_007052719.1 | XM_012541253.1 |
| AB512463.1 | GQ848199.1 | XM_007053164.1 | XM_012827888.1 |
| AB519145.1 | HE856388.1 | XM_007242830.2 | XM_012862726.1 |
| AB519146.1 | HF679025.1 | XM_007252983.1 | XM_014014850.1 |
| AB519147.1 | HG970332.1 | XM_007258943.2 | XM_014014852.1 |
| AF057028.1 | HG970334.1 | XM_007570689.1 | XM_014022018.1 |
| AF170706.1 | HM756143.1 | XM_007570729.1 | XM_014192913.1 |
| AJ005038.1 | JQ353486.1 | XM_007570736.1 | XM_014192914.1 |
| AJ009931.2 | JR995219.1 | XM_007657944.1 | XM_014192915.1 |
| AP013059.1 | JV077412.1 | XM_007815189.1 | XM_014192917.1 |
| AY247033.1 | JV093095.1 | XM_007884968.1 | XM_014204146.1 |
| AY855350.1 | JV105615.1 | XM_007884969.1 | XM_014204149.1 |
| AY856481.1 | U41661.1 | XM_008177504.1 | XM_014204150.1 |
| AY861662.1 | U51900.1 | XM_008296083.1 | XM_014468393.1 |
| BT078212.1 | XM_001633857.1 | XM_008314914.1 | XM_014468428.1 |
| CP000103.1 | XM_001782052.1 | XM_008330017.1 | XM_014572012.1 |
| CP002218.1 | XM_002153869.2 | XM_008330018.1 | XM_014689877.1 |
| CP002275.1 | XM_002166243.2 | XM_008417039.1 | XM_014986113.1 |
| CP002520.1 | XM_003039592.1 | XM_008437186.1 | XM_014986114.1 |
| CP003864.1 | XM_003448699.3 | XM_008437187.1 | XM_015006783.1 |
| CP010026.1 | XM_004080722.1 | XM_008932194.1 | XM_015015476.1 |
| CP011371.1 | XM_004080723.2 | XM_009261476.1 | XM_015015477.1 |
| CP012193.1 | XM_004206398.1 | XM_009264203.1 | XM_015040856.1 |
| CR695688.2 | XM_004552270.3 | XM_009676814.1 | XM_015040858.1 |
| CR725468.2 | XM_004565934.2 | XM_009995580.1 | XM_015385937.1 |
| CR730641.2 | XM_005468832.2 | XM_010016942.1 | XM_015400922.1 |
| FM958450.1 | XM_005468833.1 | XM_010611257.1 | XM_015608278.1 |
| FX440461.1 | XM_005725725.1 | XM_010737103.1 | XM_679508.3 |
| FX442360.1 | XM_005726061.1 | XM_010743667.1 |  |
| FX459751.1 | XM_005726062.1 | XM_010743668.1 |  |
| FX502289.1 | XM_005913798.2 | XM_010769529.1 |  |
| GAOL01014507.1 | XM_005920325.2 | XM_010769530.1 |  |

**References**

1. Macrander, J.C. Venomics of sea anemones: A bioinformatic approach to tissue specific venom composition and toxin gene family evolution. The Ohio State University: Columbus, OH, USA, 2016.
2. Rodríguez, E.; Barbeitos, M.S.; Brugler, M.R.; Crowley, L.M.; Grajales, A.; Gusmão, L.; Häussermann, V.; Reft, A.; Daly, M. Hidden among sea anemones: The first comprehensive phylogenetic reconstruction of the order Actiniaria (Cnidaria, Anthozoa, Hexacorallia) Reveals a Novel Group of Hexacorals. *PLoS ONE* **2014**, *9*, e96998.
3. Putnam, N.H.; Srivastava, M.; Hellsten, U.; Dirks, B.; Chapman, J.; Salamov, A.; Terry, A.; Shapiro, H.; Lindquist, E.; Kapitonov, V.V.; et al. Sea anemone genome reveals ancestral eumetazoan gene repertoire and genomic organization. *Science* **2007**, *317*, 86–94.
4. Stefanik, D.J.; Lubinski, T.J.; Granger, B.R.; Byrd, A.L.; Reitzel, A.M.; DeFilippo, L.; Lorenc, A.; Finnerty, J. R. Production of a reference transcriptome and transcriptomic database (EdwardsiellaBase) for the lined sea anemone, *Edwardsiella lineata*, a parasitic cnidarian. *BMC Genom.* **2014**, *15*, 71.
5. Macrander, J.; Broe, M.; Daly, M. Tissue-specific Venom composition and differential gene expression in sea anemones. *Genom. Biol. Evol.* **2016**, evw155.
6. Macrander, J.; Brugler, M.R.; Daly, M. A RNA-seq approach to identify putative toxins from acrorhagi in aggressive and non-aggressive *Anthopleura elegantissima* polyps. *BMC Genom.* **2015**, *16*, 221.
7. Baumgarten, S.; Simakov, O.; Esherick, L.Y.; Liew, Y.J.; Lehnert, E.M.; Michell, C.T.; Li, Y.; Hambleton, E.A.; Guse, A.; Oates, M.E.; et al. The genome of *Aiptasia*, a sea anemone model for coral symbiosis. *Proc. Natl. Acad. Sci. USA* **2015**, *112*, 11893–11898.
